# Prevalence and determinants of hypertension and associated cardiovascular risk factors: data from a population-based, cross-sectional survey in Saint Louis, Senegal

**DOI:** 10.5830/CVJA-2013-030

**Published:** 2013-06

**Authors:** Soulemane Pessinaba, Alassane Mbaye, Grâce-À-Dieu Yabeta, Cheikh Tidiane Ndao, Habibou Harouna, Dior Diagne, Bouna Diack, Moussa Kane, Abdoul Kane, Adama Kane, Mouhamadou Bamba Ndiaye, Malick Bodian, Maboury Diao, Maïmouna Ndour Mbaye, Khadim Niang, Jean-Baptiste Sy Mathieu

**Affiliations:** Cardiology Department, Grand Yoff Hospital, Dakar, Senegal; Cardiology Department, Grand Yoff Hospital, Dakar, Senegal; Cardiology Department, Grand Yoff Hospital, Dakar, Senegal; Cardiology Department, Grand Yoff Hospital, Dakar, Senegal; Cardiology Department, Grand Yoff Hospital, Dakar, Senegal; Cardiology Department, Grand Yoff Hospital, Dakar, Senegal; Cardiology Department, Grand Yoff Hospital, Dakar, Senegal; Cardiology Department, Grand Yoff Hospital, Dakar, Senegal; Cardiology Department, Grand Yoff Hospital, Dakar, Senegal; Cardiology Department, Aristide Le Dantec Hospital, Dakar, Senegal; Cardiology Department, Aristide Le Dantec Hospital, Dakar, Senegal; Cardiology Department, Aristide Le Dantec Hospital, Dakar, Senegal; Cardiology Department, Aristide Le Dantec Hospital, Dakar, Senegal; Internal Medicine Department, Abass NDAO Hospital, Dakar, Senegal; Department of Public Health, Chiekh Anta Diop University, Dakar, Senegal; Cardiology Department, Saint Louis Hospital, Dakar, Senegal

**Keywords:** hypertension, cardiovascular, Africa, risk factors, Senegal

## Abstract

**Background:**

The incidence of cardiovascular disease is growing worldwide and this is of major public health concern. In sub-Saharan Africa, there is a lack of epidemiological data on the prevalence and distribution of risk factors of cardiovascular disease. This study aimed at assessing the prevalence of hypertension and other cardiovascular risk factors among an urban Senegalese population.

**Methods:**

Using an adaptation of the WHO STEPwise approach to chronic disease risk-factor surveillance, we conducted a population-based, cross-sectional survey from 3 to 30 May 2010 on 1 424 participants aged over 15 years. Socio-demographic and behavioural risk factors were collected in step 1. Physical anthropometric measurements and blood pressure were documented in step 2. Blood tests (cholesterol, fasting blood glucose, and creatinine levels) were carried out in step 3.

**Results:**

The prevalence of hypertension was 46% (95% CI: 43.4–48%), with a higher prevalence in females (47.9%) than males (41.7%) (*p* = 0.015), and 50% of these hypertensive were previously undiagnosed. Mean age was 53.6 years (SD: 15.8). In known cases of hypertension, the average length of its evolution was 6 years 9 months (range 1 month to 60 years). Hypertension was significantly associated with age (*p* = 0.001), socio-professional category (*p* = 0.003), dyslipidaemia (*p* < 0.001), obesity (*p* < 0.001), physical inactivity (*p* < 0.001), diabetes (*p* < 0.001) and stroke (*p* < 0.001).

**Conclusion:**

We found a high prevalence of hypertension and other cardiovascular risk factors in this population. There is need of a specific programme for the management and prevention of cardiovascular disease in this population.

## Abstract

Hypertension (HTN) remains a major public health concern worldwide and particularly in sub-Saharan Africa.^1^-^3^ The overall prevalence of HTN worldwide is estimated to be 30% and the attributable mortality is ~30%. Lawes *et al.* reported that overall, about 80% of the attributable burden occurred in low- and middle-income economies, and over half occurred in people aged 45–69 years.^4^

In sub-Saharan Africa, the prevalence of HTN is estimated to vary between 15 and 33%.^1^ HTN is usually associated with other cardiovascular risk factors such as diabetes, dyslipidaemia and obesity.^5^ In Senegal, there is a lack of population-based epidemiological data on HTN and cardiovascular risk factors. Our study aimed at assessing the prevalence and deteminants of HTN and associated cardiovascular risk factors among an urban population in Senegal (Saint Louis).

## Methods

This study was a population-based, cross-sectional survey conducted in the city of Saint Louis (north Senegal, 250 km from the capital Dakar). It population is 190 000 inhabitants (2008 estimate) and the number of subjects over 15 years is estimated at 110 000.

Data were collected in three steps;^6^ step 1 comprised using a questionnaire to collect demographic and lifestyle data; step 2 involved measurements of height, weight, blood pressure, waist and hip circumference; and step 3 included laboratory (biochemistry) investigations. Data presented in this publication are related only to hypertension.

A list of the districts in the city was used for sampling. Each district was divided into squares and each square was subdivided into concessions (a group of households). A list of all concessions was obtained from the regional statistics office. This list was used as a sampling frame for the random selection of squares.

In each square, concessions to be visited were randomly selected and inside the concession, a household was also randomly selected. In each household, all the persons matching the selection criteria were invited to participate in the study. One hundred and twenty households were randomly selected, giving a total of 1 424 participants; 32 sets of data were not been analysed because of missing biological and/or clinical data.

Eligible criteria were age ≥ 15 years and being a resident of Saint Louis. Formal written consent was obtained. Non-consenting patients and pregnant women were not included.

Participants were involved in the survey for one day. Those with abnormal physical or laboratory findings were counselled and referred to the regional hospital as defined by the National Health reference system. Interviews, body measurements and laboratory tests were performed by nurses and clinical officers.

The survey questionnaire consisted of socio-demographic (age, gender, education, marital status), lifestyle variables (fruit and legume consumption, exposure to tobacco and alcohol, and physical activity), and medical and health history.

Physical body measurements included blood pressure (BP), height, weight, and waist circumference. Blood pressure measurements were taken using an electronic digital blood pressure machine (OMRON® M6). Three BP measurements were performed on both arms, in a seated position, legs uncrossed, after a five- to 10-minute rest. The highest BP value was recorded.

Waist circumference was measured in centimeters using a tape measure, and the measurement was made at the mid-axillary line, midway between the last rib and the superior iliac crest. Height was measured with the participant standing upright against a wall on which a height mark was made. Weight measurements were taken on a pre-calibrated weighing scale (Seca 750). Participants were weighed dressed in light clothing and barefoot.

Blood samples were analysed in a single laboratory using an automate Reflotron- Plus®. Cholesterol [total, high-density lipoprotein (HDL) and low-density lipoprotein (LDL)], triglyceride, fasting blood glucose, uric acid and creatinine levels were analysed.

Hypertension was defined as a systolic BP ≥ 140 mmHg or a diastolic BP ≥ 90 mmHg, or a documented medical history of antihypertensive treatment.^7^ Obesity was defined as body mass index (BMI) ≥ 30.0 kg/m^2^, and overweight by a BMI > 25 but < 30 kg/m^2^.

Diabetes mellitus was defined as two fasting blood glucose levels > 1.26 g/l and/or a documented medical history of diabetes or diabetes treatment. The threshold for normal values were < 2 g/l for total cholesterol, < 1.6 g/l for LDL cholesterol, > 0.4 g/l for HDL cholesterol, and < 1.5 g/l for fasting triglycerides.

Physical inactivity was defined as the absence of daily physical activity or the presence of physical activity lasting less at 150 minutes per week. Abdominal obesity was defined according to NCEP, with a waist circumference greater than 102 cm in men and 88 cm in women.

Ethics committee approval to undertake the survey was in accordance with national and local regulations. Written, signed consent was obtained for each of the patients included. The study was conducted in accordance with the Helsinki II Declaration.

## Statistical analysis

Data recorded in the standard questionnaire were double checked by external monitor and double-entered using Epi Data software. Entered data were cleaned and analysed by an experienced biostatistician using Epi info version 3.5.1 software.

Binary variables were described by their proportion and continuous variables by means and standard deviation (SD). Pearson and Yates (when appropriate) chi-square test were used for the comparison of qualitative variables and Student’s *t*-test for the comparison of quantitative variables between groups. A logistic regression model was built with variables associated with hypertension. Age and gender were forced into the final model. The results were statistically significant if *p* < 0.05.

## Results

**Fig. 1.**
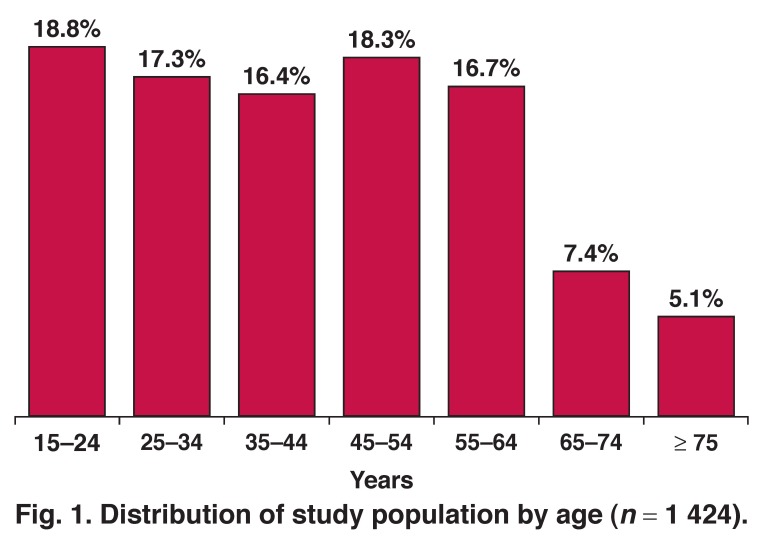
Distribution of study population by age (*n* = 1 424).

We recruited 1 424 participants (983 female, 69%). Mean age was 43.4 years (SD: 17.8), (range 15–96 years); 70.8% were < 55 years and 87.5% were < 65 years. Fig. 1 shows the distribution of the population by age. Table 1 shows the characteristics of the enrolled population and Table 2 shows the prevalence of various cardiovascular risk factors.

**Table 1.** Characteristics Of The Study Population (*n* = 1 424)

	*Female*	*Male*	*Total*	*p*
Sample size	983	441	1424	
Age (years), mean (SD)	44.2 (17.2)	41.7 (18.9)	43.4 (7.8)	0.016
Weight (kg), mean (SD)	71.7 (17.9)	67.6 (13.6)	70.5 (16.7)	< 0.001
Height (cm), mean (SD)	163.3 (8.3)	174.9 (8.5)	166 (9.9)	< 0.001
Waist circumference (cm), mean (SD)	87.4 (16.5)	81.2 (46.8)	84.6 (15.9)	0.0003
Systolic BP (mmHg), mean (SD)	131.1 (28.7)	131.9 (22.3)	131.2 (27.8)	0.893
Diastolic BP (mmHg), mean (SD)	86.7 (24.5)	82.4 (22.4)	85.4 (22.4)	0.0001
BMI (kg/m^2^), mean (SD)	27 (7.2)	22.1 (16.2)	25.5 (6.7)	< 0.001

SD: standard deviation

**Table 2.** Prevalence Of Cardiovascular Risk Factors In The Studied Population (*n* = 1 424)

*Risk factors*	*Prevalence, % (95% CI)*
Hypertension	46 (43.4–48.6)
Abdominal obesity	33.2 (30.8–35.7)
Obesity (BMI > 30 kg/m^2^)	23 (18.1–28.2)
Tobacco smokers	5.8 (4.7–7.2)
Physical inactivity	44.4 (40.2–49)
Diabetes	10.4 (8.9–12.1)
Raised cholesterol (> 2 g/l )	36.3 (33.8–38.9)
Raised LDL cholesterol (> 1.6 g/l )	20.6 (18.5–22.8)
Low value of HDL cholesterol	41.9 (39.4–44.5)
Metabolic syndrome	15.8 (14–17.8)

BMI: body mass index, CI: confidence interval.

**Fig. 2.**
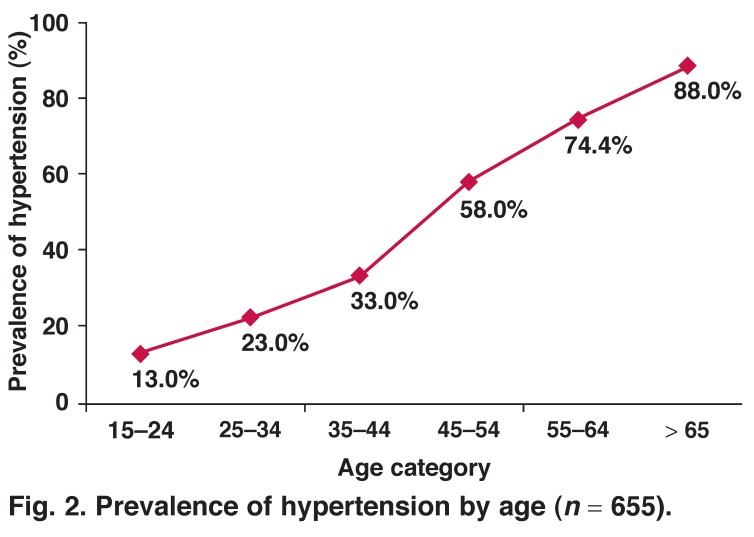
Prevalence of hypertension by age (*n* = 655).

Six hundred and fifty-five participants had HTN, giving a prevalence of 46.0% (95% confidence interval: 43.4–48.6%). Among these 655 cases, 327 (50%) were previously undiagnosed. HTN was more frequent in females [47.9% (44.8–51.1%)] than in males [41.7% (37.1–46.5%), *p* = 0.015, OR = 1.29 (1.02–1.62)]. The mean age was significantly higher in the hypertensive participants (53.6, SD: 15.8 years) than in non-hypertensive participants (34.7 years, SD: 14.5, *p* < 0.001). The prevalence of HTN increased with age (*p* = 0.001) (Fig. 2). Mean duration of HTN was 6.9 years (range: 1 month – 60 years).

Among HTN participants, mean systolic BP was 136 mmHg and mean diastolic BP 88 mmHg. Grade 1 HTN was more frequent (48%) than grade II (25%) and grade III (27%). HTN tended to be more frequent in participants who had primary school level education (42.1%) than in those who had higher levels of education (28.4%, *p* = 0.18). Table 3 shows the distribution of hypertension according to socio-professional category. There was a statistically significant relationship between hypertension and the different socio-professional categories, except for selfemployed, privately employed and volunteer participants (*p* = 0.0031).

**Table 3.** Prevalence Of Hypertension In Socio-Professional Category

	*Number*	*Hypertension (%)*	p
Official	71	36.6	1
Private	72	25	0.13
Self employed	496	48.2	0.06
Volunteer	9	22.2	0.39
Housewife	528	50.9	0.023
Student	130	10	< 0.001
Unemployed	35	57.1	0.045
Retired	83	81.9	< 0.001

Diabetes was detected in 16.5% (13.8–19.6%) of the participants with HTN and in 5.2% (3.8–7.1%) of participants without HTN [*p* = 0.023, OR = 0.32 (0.21–0.47)]. Moreover, HTN was more frequent in participants with diabetes [73% (65.1–79.9%)] than in those without diabetes [43% (40.1–45.6%), *p* < 0.0001, OR = 3.59 (2.46–5.25)].

Other risk factors associated with HTN were dyslipidaemia in 71.1% (67.5–74.6%) of participants with HTN versus 59% (55.5–62.5%) in non-HTN participants (*p* < 0.001), physical inactivity [48.5% (43.9–52.1%) vs 40.2% (36.3–44.5%), *p* < 0.001] and abdominal obesity [47.3% (43.5–51.2%) vs 21.2% (18.4–24.3%), *p* < 0.001].

**Fig. 3.**
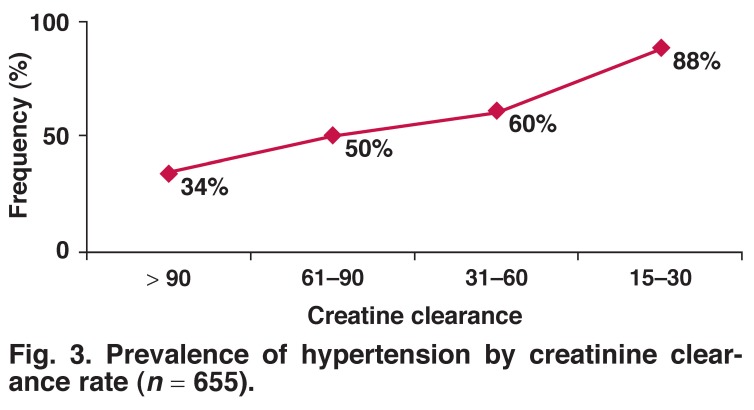
Prevalence of hypertension by creatinine clearance rate (*n* = 655).

HTN was more frequent in the case of a past history of smoking (50.8%) (41.8–59.7%) than in passive exposure (44.8%) (40.9–48.8%) and cigarette users (33.7%) (23.7–44.9%). A medical history of stroke was more frequent in participants with HTN (2.7%) (1.7–4.4%) compared with those without HTN (0.5%) (0.2–1.4%) (*p* < 0.001). HTN was correlated with the creatinine level (*p* < 0.05) (Fig. 3). The mean clearance rate of creatinine gradually decreased with the duration of hypertension (Fig. 4) (*p* = 0.26).

## Discussion

In order to gather data on the frequency of HTN and associated risk factors in urban Saint Louis residents, we carried out a population-based, cross-sectional survey with a methodology closed to the WHO STEPwise approach. We found a significant increase in the prevalence of HTN.

A previous study performed in the same region in 1970 found a prevalence of 4.9% in a rural population, whereas the prevalence was 7% in an urban population. Even though the methodology (HTN if BP ≥ 160/95 mmHg) in this study was not similar to ours, our results suggest a significant increase in the prevalence of HTN since 1970.^8^ Moreover, Kane *et al*. in 1995 found a prevalence of 20.2% with a methodology very similar to ours.^5^ In the sub-Saharan African region, two studies have reported a median prevalence of 28%, with a regional variation ranging from 15 to 38.6%.^1^,^9^ Changes in lifestyle may be the major factor leading to this increasing prevalence of HTN and other cardiovascular risk factors.^9^-^11^

While we have not found significant associations between HTN and level of education, it should be noted that previous studies found such an association. The ENNS trial found than HTN was twice as frequent in people with a primary level of education than in those who had secondary or postgraduate levels of education. This difference was higher in women: the risk of HTN was four-fold higher in less-educated women than in those with higher levels of education.^2^ The same observation was made in Brazzaville, Congo.^9^

The association between HTN and low socio-econonomic conditions is well described in studies conducted in low-income countries. The lower the socio-economic income, the higher is the probability of having HTN.^12^,^13^

In our population sample, women were more represented than men. This could have been related to the observation that women were more likely to be at home at the time the study team visited than men, who were involved in economic activities outside the home. Additionally, men were more inclined to decline participation in the survey. This observation was noticed by other authors in this kind of population-based survey.^14^

We found a predominance of HTN in women. This observation was previously reported in the CONSTANT trial in Guadeloupe (37.3 vs 33%) and Tunisia (36 vs 25%).^12^,^13^ This is in contradiction with the predominance of HTN found in males, reported in many epidemiological surveys.^2^,^14^ Some authors have suggested that women are protected from HTN up to menopause.

In our study, obesity and inactivity were significantly more frequent in women than men, and females were older than males. This could explain the predominance of HTN in the women. We also noted a significantly higher diastolic blood pressure in women than in men, for which we did not find an explanation, except that the women may have had more risk factors.

Regarding other risk factors, we found that age correlated with the prevalence of HTN. This was previously noted in Algeria and France.^9^,^15^ Obesity accounted for 11 to 25% of HTN and prevention studies have reported that a decrease of 1 kg of body weight led to a decrease of 1.1/0.9 mmHg in BP.^16^-^18^ The meta-analysis of Whelton (54 randomised clinical trials) reported a decrease of 3.8/2.9 mmHg in people with regular aerobic physical activity; the highest decrease was found in hypertensive subjects (4.9/3.7 mmHg).^17^

Obesity and physical inactivity are known to be risk factors for the onset of diabetes, HTN and other cardiovascular diseases. The review of Sowers showed that HTN was twice as frequent in patients with diabetes than in those with normal glycaemia. Additionally, Sowers reported an increase in the risk of diabetes in HTN patients compared to non-hypertensives.^17^ Dussol found that HTN was present in 80% of type 2 diabetes patients.^19^

We noticed a lower prevalence of HTN in participants who reported tobacco smoking. Nebie et al. reported a prevalence of 23% of HTN in smokers.^20^ The association between tobacco usage and HTN is still controversial and a possible confounding effect of both alcohol usage and overweight is being assumed.^21^ The association of HTN with other cardiovascular risk factors contributes to increase the global cardiovascular risk of patients.

The results showed a higher prevalence of hypertension with worsening creatinine clearance rates. This was probably a consequence of hypertension, as shown by the decrease in creatinine clearance rate with the duration of hypertension.

## Conclusion

This population-based survey is the first performed in Senegal. It was intended to serve as a baseline situation for other surveys locally or at a national level. We found a high prevalence of hypertension associated with other cardiovascular risk factors such as diabetes, obesity, inactivity and dyslipidaemia. The majority of participants were not aware of their condition.

Nationwide surveys are needed to better assess the burden of cardiovascular disease in this population. This will help authorities to formulate and implement adequate strategies to control hypertension and the emerging epidemic of non-communicable diseases.
